# Genetic dissection of serum pro-neurotensin suggests potential causal impact on brain structure

**DOI:** 10.1016/j.ebiom.2025.106105

**Published:** 2026-01-10

**Authors:** Jana Breitfeld, Katrin Horn, Akhil Velluva, Frauke Beyer, Janne Pott, Ronny Baber, Michael Stumvoll, Diana Le Duc, Veronica Witte, Alice Giontella, Olle Melander, Peter Kovacs, Markus Scholz, Anke Tönjes

**Affiliations:** a Department of Medicine III, Division of Endocrinology, Nephrology and Rheumatology, University of Leipzig, Leipzig, Germany; b Institute for Medical Informatics, Statistics and Epidemiology, University of Leipzig, Leipzig, Germany; c LIFE Research Center for Civilization Diseases, University of Leipzig, Leipzig, Germany; d Department of Evolutionary Genetics, Max Planck Institute for Evolutionary Anthropology, Leipzig, Germany; e Rudolf Schönheimer Institute of Biochemistry, Medical Faculty, University of Leipzig, Germany; f Cognitive Neurology, University Medical Center Leipzig, and Max Planck Institute for Human Cognitive and Brain Science, Leipzig, Germany; g Bordeaux Population Health Research Center, University of Bordeaux, Inserm, UMR, 1219, Bordeaux, France; h University of Cambridge, MRC Biostatistics Unit, Cambridge, United Kingdom; i Institute of Laboratory Medicine, Clinical Chemistry and Molecular Diagnostics, University of Leipzig, Leipzig, Germany; j LIFE, LIFE Leipzig Research Center for Civilization Diseases, University of Leipzig, Leipzig, Germany; k Deutsches Zentrum für Diabetesforschung, Neuherberg, Germany; l Helmholtz Institute for Metabolic, Obesity and Vascular Research (HI-MAG) of the Helmholtz Center Munich at the University of Leipzig and University Hospital Leipzig, Leipzig, Germany; m Institute of Human Genetics, University Medical Center Leipzig, Leipzig, Germany; n Department of Clinical Sciences, Lund University, Malmö, Sweden; o Lund University Diabetes Center, Lund University, Malmö, Sweden; p Department of Internal Medicine, Skåne University Hospital, Malmö, Sweden; q Metabolic Center, Region Skåne, Malmö, Sweden

**Keywords:** Obesity, Brain magnetic resonance imaging, Pro-neurotensin, Genome-wide association study, Mendelian randomisation

## Abstract

**Background:**

Pro-neurotensin (pro-NT) is the stable circulating precursor of neurotensin (NT), a neuropeptide expressed mainly in the central nervous system and small intestine that regulates key physiological processes like fatty acid absorption in the gut and suppresses appetite via central mechanisms. Studies in NT-deficient mice and humans implicate NT in obesity and insulin resistance, highlighting its role in metabolic regulation.

**Methods:**

To explore the genetic determinants of circulating pro-NT and its causal relationships with obesity and brain phenotypes, we conducted a genome-wide meta-analysis of serum pro-NT levels in 10,096 individuals of European ancestry across four independent cohorts. We further examined causal effects of pro-NT on brain structures and function using Mendelian Randomisation (MR) and analysed brain magnetic resonance imaging (MRI) data from a subset (N = 1090) of the LIFE-Adult cohort.

**Findings:**

Three genome-wide significant loci associated with serum pro-NT were identified on chromosomes 4 (rs6822751), 11 (rs41392245), and 12 (rs2723889). MR analyses revealed causal links between elevated pro-NT and structural variation in selected subcortical brain regions, notably the pallidum and brainstem. MRI analyses in the LIFE-Adult subset showed reduced reward network coherence in alleles linked to higher pro-NT levels, suggesting a potential neural mechanism contributing to obesity.

**Interpretation:**

These findings suggest a potential causal relationship between serum pro-NT levels and variance in structural brain phenotypes that could be implicated in obesity.

**Funding:**

See Acknowledgements. Key funding bodies: Deutsches Zentrum für Diabetesforschung (DZD, Grant: 82DZD06D03) to MSt; European Union, by the European Regional Development Fund (ERDF) to RB; Swedish Foundation for Strategic Research (IRC LUDC), Swedish Research Council (SFO-EXODIAB), Swedish Research Council (AIR Lund- Artificially Intelligent use of Registers at Lund University, VR; Grant No. 2019-61406) to OM.

**Research in context**

**Evidence before this study**

Neurotensin (NT) is a 13-amino acid peptide expressed primarily in the central nervous system (CNS) and the small intestine. Tissue-specific processing may lead to different forms of NT.

In the CNS, NT acts as a neurotransmitter, regulating various processes such as body temperature, nociception, pituitary hormone secretion, and dopaminergic transmission. In response to fat intake, NT facilitates intestinal absorption of fatty acids based on the lipid content of the diet. Peripherally, NT promotes fat absorption and weight gain, whereas central NT signalling suppresses feeding and promotes weight loss.

Studies in NT-deficient mice have shown protection against obesity, hepatic steatosis and insulin resistance associated with high fat intake. A genetic study showed that a variant in the neurotensin gene is associated with circulating pro-NT levels in children with overweight and obesity. Carriers of the low-risk allele have a more favourable metabolic profile and less insulin resistance later in life. Other human studies have shown that people with obesity and insulin-resistance have elevated pro-NT levels, which may be an indicator of obesity risk in later life.

**Added value of this study**

Meta-GWAS followed by secondary analyses revealed three loci with significant associations: i) on chromosome (chr) 4—rs6822751; ii) on chr 11—rs41392245 and; iii) on chr 12—rs2723889. Mendelian Randomisation (MR) analyses using these three variants as instruments suggested a causal effect of pro-NT on pallidum brain structure.

In a subsample with magnetic resonance imaging (MRI) measurements (N = 1090), we observed that variants associated with higher pro-NT are associated with lower reward network coherence, a phenotype associated with obesity development.

This study not only revealed genetic variants associated with serum pro-NT, but also provides causal and associative connections to brain phenotypes.

**Implications of all the available evidence**

The present study improves our understanding of genetic mechanisms influencing pro-NT and its relationships to brain structure and function. Further research should investigate the underlying mechanisms to clarify whether and how (pro-NT) could serve as therapy target for obesity and metabolic disorders.

## Introduction

Pro-neurotensin (pro-NT), the stable precursor of the neuropeptide neurotensin (NT), is increasingly recognised not only for its diverse physiological roles but also as a circulating biomarker closely linked to adverse health outcomes. Elevated basal levels of circulating pro-NT have been robustly associated with an increased risk of multiple public health concerns, including obesity, metabolic syndrome, cardiovascular disease, and even mortality, as demonstrated in several large-scale epidemiological studies.^1^, ^2^, ^3^ These findings highlight that pro-NT is more than a participant in biological pathways; its basal plasma concentration serves as a critical indicator of disease risk and progression.

Biologically, NT modulates key metabolic and neurological processes both centrally—in the brain, where it regulates appetite, neurotransmitter release, blood pressure, heart rate, and pain perception—and peripherally—in the gut, influencing fatty acid absorption and gastrointestinal motility. These multifaceted actions have positioned NT as a promising therapeutic target for metabolic disorders, cardiovascular conditions, and neuropsychiatric diseases.^4^^,^^5^ Moreover, NT's influence on stress response, neuroinflammation, cognition, and neuroplasticity underscores its involvement in the pathophysiology of mood disorders.^4^

Despite the clear clinical relevance of pro-NT, understanding the genetic underpinnings that regulate circulating pro-NT levels remains limited. Identification of genetic variants associated with pro-NT could elucidate biological pathways driving NT signalling dysregulation and offer novel drug targets for associated diseases. Prior research has identified a variant in the *neurotensin* gene (*NTS*; rs2234762) linked to lower circulating pro-NT levels in children with overweight, suggesting a genetic contribution to metabolic risk profiles and insulin sensitivity.^6^ Observational studies further support pro-NT plasma levels as predictive marker for obesity development, with variable levels reported across different stages of obesity.^7^

Building upon these insights, we conducted a comprehensive meta-analysis of four independent cohorts totalling 10,096 individuals (LIFE-Adult, Malmö Diet Cancer (MDC), Malmö Offspring Study (MOS), and the Sorbs cohort). Our analysis not only confirmed the genetic determinants of circulating pro-NT but also identified causal effects of pro-NT on brain structural and functional traits via Mendelian Randomisation (MR) approaches. To test the results with respect to their specificity to the reward network, we focussed on the cortico-striatal network as the central system to evaluate reward value and initiate reward-driven behaviour. It includes the orbitofrontal cortex and the ventral striatum as key structures to mediate between pleasure, motivation and reward processes.^8^ While both of these regions are tightly connected to other brain regions, including amygdala and hippocampus, we decided to focus on the core network here to be in line with previous publications investigating its role in obesity.^9^^,^^10^

Complementary analyses integrating human and murine gene expression data, alongside MRI-based assessments of obesity-related brain reward network connectivity, revealed pleiotropic roles for pro-NT in linking neuronal correlates with peripheral metabolic signals. These findings reinforce pro-NT as a key biomarker and potential mediator of complex interactions between metabolic and neuropsychiatric health.

## Methods

### Study design and cohorts

Four independent cross-sectional studies have been included in the present genetic meta-analysis, namely MDC,^11^^,^^12^ MOS,^13^ LIFE-Adult and the Sorbs cohort. We briefly introduce these studies presenting descriptive statistics of all studies in Table 1. Details of study-specific genotyping, phenotyping, quality control and data analysis are provided in Supplementary Table S1.

**Table 1:** Cohort description.

	MDC	MOS	Sorbs	LIFE-adult
N	4326	1721	967	3082
Sex (male/female)	2701/1625	774/947	397/570	1440/1642
Age (years)	58 ± 6	39 ± 14	48 ± 16	63 ± 11
BMI (kg/m^2^)	25.8 ± 3.9	25.7 ± 4.7	27.0 ± 5.0	27.3 ± 4.3
N (healthy, overweight, obesity)	1925/1817/584	768/623/330	352, 379, 223	969, 1394, 710
Pro-NT (pmol/l)	123.0 ± 77.8	131.0 ± 58.5	125.0 ± 56.9	115.2 ± 50.1

MDC = Malmö Diet Cancer; MOS = Malmö Offspring Study; N = number; BMI = body-mass index; pro-NT = pro-neurotensin serum level; means ± standard deviation (SD).

We analysed retrospective observational cohort data and included participants with complete genetic and phenotypic characterisation required for our regression models. No additional inclusion or exclusion criteria were applied. Because the data were pre-existing and not collected through a prospective study design, procedures such as randomisation and blinding were not applicable.

### Ethics

#### Malmö Offspring Study (MOS) and Malmö Diet Cancer (MDC)

The ethical approval for the MOS cohort was obtained from the Regional Ethics committee (REPN) in Lund, Sweden (Dnr. 2012/594). Further, linked to this, the ethical approval for re-examination of the parents (G1) in the MDC-CC cohort 2007–2012 (Dnr. 532/2006) and the original application for the MDC baseline examination (LU-51-90). The MOS biobank was also registered at the Regional and National Biobank Register, Sweden. Written informed consent from all participants was obtained.

#### Sorbs and LIFE-Adult

The Sorbs and LIFE-Adult studies have been approved by the ethics committee of the University of Leipzig, Germany (application number for the Sorbs cohort Reg.No: 088-2005 and for the adipokine genome-wide association study AZ.: 330-12-24092012; Reg. No. for LIFE-Adult: 263-2009-14122009 and 201/17-ek) and meet the ethical standards of the Declaration of Helsinki. Written informed consent from all participants was obtained.

Human genetic and transcriptomic reference data were obtained from publicly accessible repositories. GTEx v7 and GTEx v8 data were downloaded from the GTEx Portal (https://gtexportal.org) on 15 May 2020 and 9 June 2020, respectively. Human brain expression data from the Allen Brain Atlas (ABA) were retrieved from the Allen Institute for Brain Science website (https://portal.brain-map.org). Murine RNA-seq data used for the analysis of the Nts-knockout mouse model were obtained from publicly accessible repositories. Raw sequencing data for both dietary conditions (nutrient-rich and fasting) were downloaded from the Sequence Read Archive (https://www.ncbi.nlm.nih.gov/sra, SRA; project PRJNA758022). All datasets represent experimentally generated murine transcriptomic profiles.

### Malmö Diet Cancer—MDC-cohort

To screen for dietary habits in order to predict incident cancers the longitudinal prospective MDC-cohort started in the early 1990s as a screening survey of the middle-aged population of Malmö, Sweden. We here analyse data of a follow-up campaign performed in 2007–2012. 4326 participants have been included in this analysis (Table 1, Supplementary Table S1). Exclusion criteria comprised missing DNA samples or missing bio-material for pro-NT measurements.^14^

### Malmö Offspring Study (MOS)

In the Malmö Offspring Study that started in 2013, 1721 participants (39 ± 14 years) representing children and grandchildren of index participants from the first generation, examined in the Malmö Diet Cancer Study during 1991–1996 were examined. Exclusion criteria were difficulties in understanding information in Swedish. Further, participants with missing DNA or missing bio-material for pro-NT measurements were excluded.^13^ In total, 1721 individuals have been included in this study (Table 1, Supplementary Table S1).

Although relatedness exists between MDC and MOS, we analysed these cohorts separately and combined them only in the meta-analysis. This approach was chosen to avoid batch effects from pro-NT measurements or the use of different genotyping platforms. To check a possible relationship of results between MDC and MOS, we determined the correlation of Z-scores between the cohorts. This correlation was small and comparable to that observed between MDC/MOS and LIFE-Adult (all |r| < 0.01), justifying our approach (see Supplementary Figure S1).

### LIFE-adult cohort

The population-based LIFE-Adult cohort includes 10,000 inhabitants of the city of Leipzig, Germany (age-range 18–80, age/sex stratified random collection). All participants underwent a broad range of clinical examinations, interviews and standardised questionnaires which were conducted by trained study personal at the LIFE research center in the University Hospital of Leipzig (see^15^ for details).

Out of the total cohort, 3082 participants were selected for our analysis (Table 1, Supplementary Table S1).

### Sorbs cohort

Participants included in this volunteer-based study have been recruited from the self-contained population of Sorbs in Germany and extensively metabolically phenotyped between 2005 and 2007 as described elsewhere.^16^^,^^17^ The final sample set consisted of 967 participants where complete genotype, covariate and phenotype information were available (Table 1, Supplementary Table S1).

### Measurement of pro-NT

Pro-NT concentrations of MOS and MDC were measured in fasting plasma specimens that were frozen to −80 °C immediately after extraction.^14^ In LIFE-Adult and the Sorbs cohort, pro-NT was measured in fasting serum samples, also frozen to −80 °C immediately after extraction using the same method. A description of the chemiluminometric sandwich immunoassay used in all four cohorts to detect the pro-NT precursor fragment (pro-NT 1–117^18^) has been described in detail elsewhere.^3^ Means and standard deviations of pro-NT were similar across cohorts, (see Supplementary Table S1).

For the MOS and MDC cohorts, we used the same batch of reagents (RRID:AB_3086809) for each analysed cohort to measure the inter-assay variability of pro-NT. For the LIFE-Adult and Sorbs cohorts, pro-NT was measured by a company (using same reagents (RRID:AB_3086809)) that provided the following data: the inter-assay variability was ≤5%. Further internal controls for the kits were included in each run, measuring a low and a high control. The results showed variability of 2.3% and 2.4% between the single runs. Analysis of the pro-NT distributions showed good adherence to a log-normal distribution, with no signs of heterogeneity or mixture of different distributions.

To avoid potential batch effects in pro-NT measurements due to the single measurements being taken in two different laboratories to detect the plasma pro-NT precursor fragment (pro-NT 1–117), using both the chemiluminometric sandwich immunoassay the sphingotest® pro-NT Test (Sphingotec; RRID:AB_3086809), we decided not to pool samples but rather to perform a meta-analysis and to examine heterogeneity across studies.

### Statistics

#### Genetic analyses

##### Genotyping, quality control, and genotype imputation

Genotype determination occurred using the single nucleotide polymorphism (SNP) micro-arrays: Illumina Human OmniexpressExome v1.0 in MDC, Illumina GSA Beadchip in MOS, Affymetrix Gene Chip Human Mapping 500k or 1000k Array Set in the Sorbs cohort, and Affymetrix Axiom CEU1 in LIFE-Adult. Sample and SNP quality control was done in accordance to study specific criteria as specified in the Supplementary Table S1.

Genotypes were imputed on the Haplotype Reference Consortium Reference Panel (MDC and MOS) using IMPUTE4 or on the 1000 Genomes phase 3 reference using IMPUTE2 (Sorbs and LIFE-Adult), respectively. Genotype data were translated to forward strand annotation using NCBI b37 (hg19) coordinates.

##### Single study genome-wide association study (GWAS)

Single study genetic analyses were performed according to a common analysis plan shared with the study analysts (OM; MSch; AT). Pro-NT values were log-transformed to approximate normal distribution. Analyses were performed by linear regression modelling assuming an additive genetic model and adjusting for age and sex. Beta-coefficients of the genetic main effect were used as primary statistics and tested against 0 (two-sided test). Association analyses have been performed using PLINK 2.0.

Quality control for single study GWAS was done centrally using the R package EasyQC vs 9.2^19^ We excluded SNPs with missing alleles or statistics (e.g. beta estimates, imputation quality score), mismatched alleles or mismatched chromosomal positions with respect to the reference. Further, SNPs were filtered for minor allele frequency (MAF) >1%. Genotyped SNPs were selected by call rate ≥ 0.8 and P-value of Hardy-Weinberg disequilibrium P > 10^−6^. Imputed SNPs were filtered by imputation quality score >0.5. For all SNPs the allele frequency was compared to that of the 1000Genomes phase 3 version 5 reference and variants were discarded where the difference was >20%. Finally, study results were harmonised to the same reference alleles. Number of quality-controlled SNPs per study is presented in Supplementary Table S1. Genomic control inflation factors λ were determined for each single study GWAS. Respective test statistics were corrected by Genomic control if λ > 1.

##### Meta-GWAS and hit annotation

To summarise single-study GWAS, fixed effects inverse variance meta-GWAS of study-specific Beta-coefficients was performed. I^2^ values were calculated to assess heterogeneity of study results. SNPs with I^2^ > 0.75 or SNPs for which association statistics of only one study was available were discarded. The random effects model resulted in deflated test statistics (λ = 0.9) indicating loss of power. Given the low heterogeneity observed across studies, the fixed-effect model was deemed appropriate for our data. A P-value (two-sided Z-test of meta-effect) of 5 × 10^−8^ was considered as genome-wide significant. To assess ancestral heterogeneity between studies, we performed Meta-regression using the tool MR-MEGA v.0.2.^20^ Analysis results were checked for genome-wide significant variants for validation of our genome-wide analysis meta-analysis (GWAMA) findings and for ancestral heterogeneity of the studies by checking the P-value ($\chi^{2}$ test) of the respective estimates (P_anc_). To rule out secondary associations driven by body-mass index (BMI), we conducted conditional analyses of the genome-wide significant loci using BMI summary statistics from Loya et al.^21^

Annotation of GWAMA results was done using an in-house workflow as described in detail elsewhere.^22^ Briefly, linkage disequilibrium (LD) between markers was calculated on 1000 Genomes Phase 3, version 5 reference panel for European samples. Priority pruning of the top-list was performed by the assumption that a variant is tagged when it is in LD (r^2^ ≥ 0.1) to a tagging-SNP of stronger association with the analysed phenotype. Loci were defined by cytobands. Further, all genes within a distance of 50 kilobases (kb) and up to four genes within a distance of 250 kb to a SNP according to Ensembl^23^ were reported as candidate genes due to proximity. SNP annotation was performed by further resources including known trait associations via LD-based lookup (r^2^ ≥ 0.3) of the GWAS Catalogue,^24^ by expression quantitative trait loci (eQTLs) based on LD-based lookup (r^2^ ≥ 0.3) of Genotype-Tissue Expression (GTEx) V7^25^ and (updated) own data^26^ and by different deleteriousness scores including Combined Annotation Dependent Depletion (CADD)^27^ and RegulomeDB.^28^ Nearby genes and eQTL genes per locus were further annotated by pathway enrichment analysis checking for over- or under-representation (hypergeometric test). We used multiple pathway ontologies for enrichment analysis because each provides complementary biological insights. Specifically, GO (Gene Ontology) focuses on the molecular functions, biological processes, and cellular components of gene products; KEGG maps genes to well-defined metabolic and signalling pathways; Reactome provides expert-curated pathway annotations with detailed molecular interactions; and DOSE focuses on disease ontology, linking genes to disease mechanisms. Using these complementary resources allowed us to annotate our findings in a more comprehensive and biologically meaningful way.

##### Determination of independent variants and credible sets

Conditional and joint analysis (COJO) was performed to detect independent variants per locus. We applied the software Genome-wide Complex Trait Analysis (GCTA), version 1.92.0beta2 for this purpose.^29^ The genetic dataset of LIFE-Adult was used as LD reference (N = 7669). GCTA function SLCT was applied to identify independent variants by a step-wise forward selection. After identification of independent SNPs we calculated their explained variance using the formula r^2^ = β^2^/(β^2^+N × se(β)^2^).^30^ Here, β denotes the fixed meta-effect of the beta estimates of additive SNP effects of the single study linear regression analyses conditioned to all other independent variants if applicable, se (β) it's standard error and N the total sample size. Total explained variance was determined by summing up the explained variances of single independent SNPs.

For each independent variant, we constructed credible sets to identify likely causal variants. Specifically, we considered all SNPs within ±500 kb of the lead SNP together with their effect estimates and standard errors. Approximate Bayes Factors (ABFs) were calculated using the R package „*gtx“*. The prior distributions of standard deviations were derived empirically by calculating the difference between the 97.5th and 2.5th percentiles of SNP effect sizes at each locus. ABFs were then used to compute the posterior probability (PP) of each variant being causal. Variants were ranked in descending order of their PP, and the cumulative probability was determined. A 99% threshold was applied to define the 99% credible set, i.e. the minimal set of variants with a combined PP of 99%, which is expected to contain the causal variant. Finally, variants within each credible set were annotated as described above and used to assign candidate genes.

##### Combined Meta-GWAS with publicly available data

Pietzner et al. study^31^ analysed semi-quantitative levels of NT and other proteins for genetic associations. Summary statistics were downloaded, harmonised to the same effect allele and used to corroborate our GWAMA hits by combined meta-analysis. The “metagen” function of the R library “meta” was used for this purpose with default settings.

### Secondary analysis for candidate gene assignment

#### MetaXcan analysis

To prioritise candidate genes, we applied the MetaXcan approach,^32^ testing the genetically regulated gene expression for association with NT using the summary statistics from our meta-GWAS. Gene expression prediction models were downloaded from the MetaXcan github repository (https://github.com/hakyimlab/MetaXcan; 06/09/2021), trained on GTEx v8 data. To use these models, we lifted our data from hg19 to hg38 using the GWAS Summary Statistics harmonisation tool and imputed missing variants as suggested by the MetaXcan developers (https://github.com/hakyimlab/summary-gwasimputation.). We tested selected candidate genes based on the results of our GWAMA, namely neurotensin (*NTS*), stabilin 2 (*STAB2*), alpha-protein kinase 1 (*ALPK1*), calcium binding protein 39 like (*CAB39L*), and tissues considered functionally relevant for NT (13 brain tissues, 2 colon tissues, liver, and whole blood). For mucin 2 (*MUC2*), no genetic models were available. Thus, gene-based associations could not be calculated for this gene. To correct for multiple testing, we applied false discovery rate (FDR) correcting on analysed tissues per gene.

#### Co-localisation analysis of gene-eQTL, sQTL and mQTLs

We investigated the overlap of causal variants between pro-NT associations and expression quantitative trait loci (QTLs), splicing QTLs (sQTLs), and methylation QTLs (mQTLs). For this analysis, we utilised eQTL data from the latest GTEx V8 release.^33^ sQTL data from Qi et al.^34^ and mQTL data from Hatton et al.^35^ for samples with European ancestry. To harmonise the genome builds between GTEx (hg38) and our meta-GWAS (hg19), we lifted the GTEx eQTLs to hg19 using the SNP lookup table provided by GTEx. For each independent genome-wide significant SNP in our meta-GWAS, we considered the nearest annotated genes within a ±250 kb window and genes that are regulated in cis (cis-eQTLs with r^2^ ≥ 0.3 with the index variant). The data for the sQTL and mQTL analysis was extracted with SMR (vs. 1.4.0) for the three independent loci.

For the co-localisation analysis,^36^ we focused on the intersection of QTLs available in summary statistics and those included in our meta-analysis. A PP of ≥75% for hypothesis (H)4 (shared signal) was considered strong evidence for co-localisation of pro-NT and the corresponding gene expression signals. In contrast, a PP of ≥75% for H3 (independent signals) was interpreted as evidence for independent signals.

#### LD-score regression analysis

LD-score regression allows estimation of heritability for single traits and genetic correlation between two heritable traits.^37^ This method was implemented in the tool LDSC v1.0.1,^38^ which we applied to first estimate the SNP-wide heritability of NT, and then, its genetic correlations with traits of subcortical brain volume.^39^ We would like to acknowledge that this GWAS provides publicly available summary statistics for seven pre-defined subcortical brain regions, which formed the basis for our analyses.

#### Bioinformatic and transcriptomic analyses

To functionally interpret our GWAMA findings and explore the pleiotropic role of pro-NT, we conducted a series of bioinformatic and transcriptomic analyses across both human and murine public datasets.

1. Co-expression Analysis in Human Tissues

We performed co-expression association analysis to investigate potential transcriptomic co-regulation involving *NTS*. Tissue-specific gene expression profiles with transcripts per million (TPM) > 1 were retrieved from GTEx V7, covering 54 non-diseased tissue types from approximately 1000 individuals. Tissues expressing *NTS* were selected for further analysis and included brain regions (amygdala, hippocampus, hypothalamus, substantia nigra) and the small intestine (terminal ileum).

In each selected tissue, we computed Pearson correlation coefficients between *NTS* expression and all other expressed genes. Genes ranking in the top 5% of correlation coefficients were retained for downstream analyses.

For brain tissues, we investigated whether *NTS*-co-expressed genes were enriched during specific neurodevelopmental stages. This was done using the ABAEnrichment R package, which leverages expression data from the Allen Brain Atlas (ABA). The analysis tested for overrepresentation of *NTS*-correlated genes in defined brain regions and developmental stages.

In the small intestine, functional annotation of the top 5% *NTS*-correlated genes (N = 867) was performed using Gene Ontology (GO) enrichment analysis via a hypergeometric test.

2. Analysis of *Nts*-Knockout Mouse Model

To further characterise the physiological role of *NTS*, we analysed RNA-seq data from a *Nts*-knockout mouse model under two dietary conditions: nutrient-rich and fasting (SRA project PRJNA758022). Raw sequencing reads were quality-checked, mapped to the mouse genome (GRCm38/gencode.vM23) using STAR, and deduplicated using samtools. Gene-level counts were obtained using HTSeq-count, and genes with <10 reads across all samples were excluded.

Differential gene expression analysis was conducted using DESeq2, comparing both experimental arms (nutrient-rich and fasting) to a common control group (*Nts*+/+). Nominally significant DEGs were subjected to GO enrichment analysis. To assess shared regulatory patterns, we identified DEGs that were directionally consistent across both conditions (i.e. consistently up- or down-regulated). GO enrichment was performed on these genes using over-representation analysis, with significance defined as family-wise error rate (FWER) ≤ 0.05 based on 1000 random permutations.

3. Cross-species Comparison of *NTS*-correlated Genes

To evaluate the conservation of *NTS*-related transcriptomic signatures between humans and mice, we compared the top 5% *NTS*-correlated genes in human small intestine (N = 867) with their murine orthologues (N = 768, per Ensembl Release 110, GRCm39). These were cross-referenced with DEGs from the *Nts*-knockout mouse model under both nutritional conditions. Overlap significance was assessed using over-representation analysis, with a background of 22,198 mouse protein-coding genes.

Additionally, we focused on two genes identified in the GWAMA results, *MUC2* and *STAB2*, due to their known roles in gastrointestinal physiology. These genes were further examined within the context of small intestinal gene networks to explore potential links to the peripheral actions of pro-NT.

#### Correlation analyses for lipid parameter

To address a possible correlation between pro-NT serum level and the lipid parameter high-density lipoprotein cholesterol (HDL-chol), low-density lipoprotein cholesterol (LDL-chol), triglycerides (TG) and total cholesterol we conducted simple Pearson correlation analyses in the available individual-level data of LIFE-Adult (N = 2958) and the Sorbs cohort (N = 967). To achieve normal distribution pro-NT and TG were log transformed.

#### Mendelian Randomisation (MR)

Due to a significant genetic correlation, we investigated a possible causal link between pro-NT and seven selected subcortical brain regions showing the strongest genetic correlation with pro-NT.^39^ The instruments of pro-NT concentrations were retrieved from the present meta-analysis, i.e. comprised the three independent hits identified in our COJO analysis. These SNPs represent strong instruments of pro-NT as demonstrated by F-statistics larger than 10 (see Supplementary Table S2), i.e. the first MR pre-condition was fulfilled.

Summary statistics of genetic associations of our pro-NT instruments with seven different subcortical brain volumes were retrieved from the study of Satizabal et al.^39^ As specified there, the GWAS models assumed additive genetic effects and were adjusted for age, sex, and total intracranial volume. However, as the study only reported Z-scores, we calculated beta estimates and standard errors using the formula proposed by Teumer et al.^40^ It revealed that the SNPs were not directly associated with globus pallidus volume so that any direct effects of the variants on globus pallidus volume not mediated via pro-NT are unlikely, i.e. the second MR pre-condition can be assumed.

Regarding possible associations with confounders of the relationship between pro-NT and globus pallidus volume, we observed pleiotropic effects at our loci with respect to the following traits: von Willebrand factor, myopia, BMI and photic sneeze reflex. Instrument rs6822751 (4q25) was in some LD (r^2^ = 0.76) with a variant found to be associated with BMI in a large genome-wide meta-analysis. However, in our LIFE-Adult study this association was very small (P = 0.26, two-sided t-test) explaining only 0.04% of total trait variability. Thus, we conclude that this potential horizontal pleiotropic effect is irrelevant for our situation. From a biological point of view and based on a co-citation analysis of the literature, we also consider it unlikely that the other traits serve as confounders of the pro-NT/globus pallidus volume association. Nevertheless, to cope with possible remaining horizontal pleiotropy, we decided to apply robust MR methods in a sensitivity analysis.

We used the inverse-variance weighted (IVW) method to combine the effects of the three instruments^41^ and assessed its heterogeneity with Cochran's Q. To investigate possible pleiotropic effects, we used MR-Egger, penalised and robust IVW as implemented in the R-package “MendelianRandomization” (vs 0.7.0)^42^ and compared it to our main findings.

### Outcome validation

#### MRI diffusion-weighted (DWI) reward network analysis

We further tested the association of our three GWAS findings with the connectivity of the reward network, a brain network important for reward processing and shown to be altered in obesity.^10^^,^^43^^,^^44^

We investigated DWI based connectivity of the reward network between major hubs of the network in LIFE-Adult participants with available MRI data according to Beyer et al.^9^ Briefly, head MRI was acquired at 3 T using anatomical and diffusion-weighted scans (60 directions) and a white matter connectome was reconstructed according to in-house pre-processing routines. These included tensor-fitting and a deterministic pathway tractography,^45^ which was computed for 82 (sub)cortical regions as nodes and connectivity weights between the regions as edges. Two types of connectivity weights were assessed (i) total number of connecting streamlines touching both regions (NOS) and (ii) mean fractional anisotropy (FA) across voxels included in these streamlines. Graph metrics (connectivity strength (CS) and clustering coefficient (CC)) were calculated using the Brain Connectivity Toolbox in Matlab 9.7 (2019b). For the reward network, bilateral, lateral and medial orbitofrontal cortex, caudate, putamen, and accumbens were used as nodes and their respective structural (NOS and FA strength) connectivity metrics as edges prior to normalisation of the measures by the corresponding whole brain connectivity metrics.^10^ This resulted in the following reward network metrics: FA CS (indicating connectivity strength based on microstructural properties), FA CC (indicating clustering within the network based on microstructural properties), NOS CS (indicating connectivity strength based on the macroscopic number of streamlines between the regions), and NOS CC (indicating clustering within the network based on the number of streamlines between regions). For all metrics, a higher value represents stronger connectivity of the network relative to the whole-brain network. Outliers were defined as values higher than (3rd quartile + 2 × interquartile range (IQR)) or lower than (1st quartile − 2 × IQR). This led to the exclusion of two participants with bad data quality (excessive head motion and large ventricles).

For a total of N = 1090 samples, combined high-quality genotype and MRI data are available. To test associations of our variants with these phenotypes, we performed analyses of additive and dominant effects using linear regression analysis adjusting for age and sex.

We performed the same analyses using the global whole-brain metrics as outcome.

**Fig. 1:**
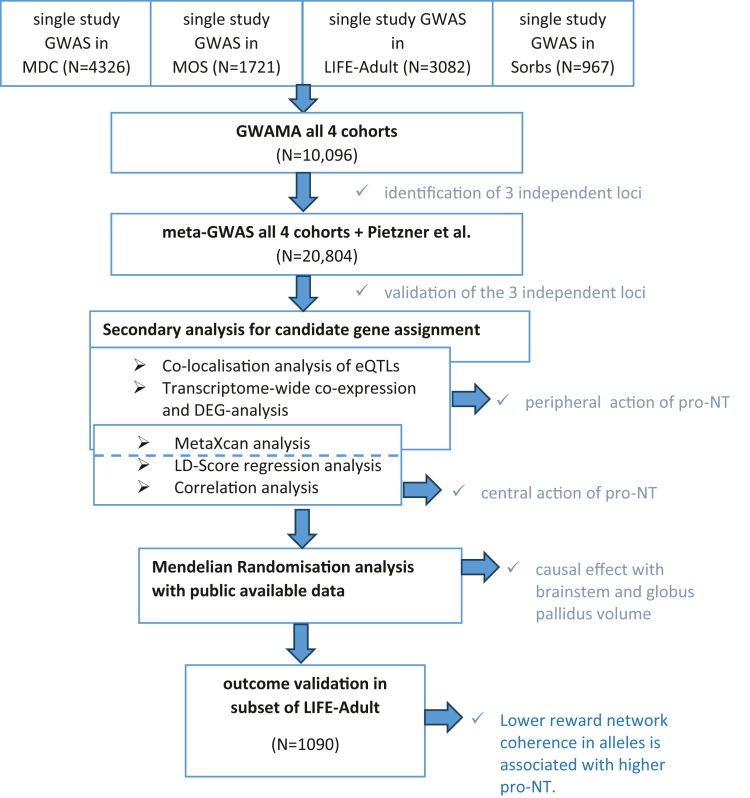
**Flow chart of pro-neurotensin analyses.** The main outcomes of the individual steps that lead to a better understanding of the subsequent step are highlighted. GWAS = genome-wide association study; GWAMA = genome-wide association meta-analysis; eQTLs = expression quantitative trait loci; DEG = differentially expressed genes; LD = linkage disequilibrium. Reference: Pietzner et al.^31^

A flow chart providing an overview of the analyses conducted is presented in Fig. 1.

### Role of funders

The funders had no role in study design, data collection, data analyses, interpretation, or writing.

## Results

### GWAMA for pro-NT identifies three independent loci

**Fig. 2:**
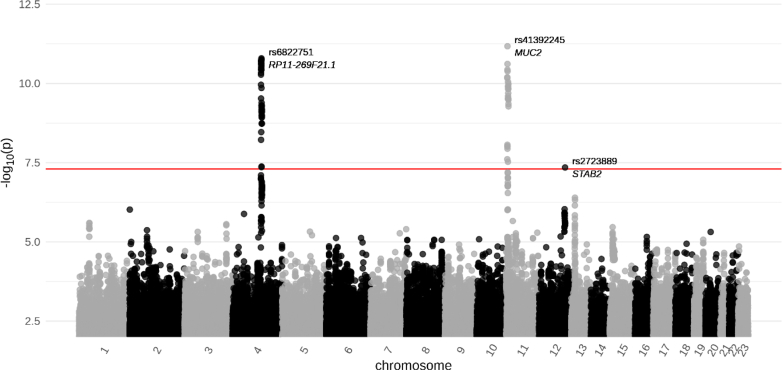
**Manhattan plot of pro-neurotensin (pro-NT) meta-genome-wide association study in four independent cohorts (N** = **10,096).** The line corresponds to the genome-wide statistical significance (P < 5 × 10^−8^). MUC2 = mucin 2; STAB2 = stabilin 2.

**Fig. 3:**
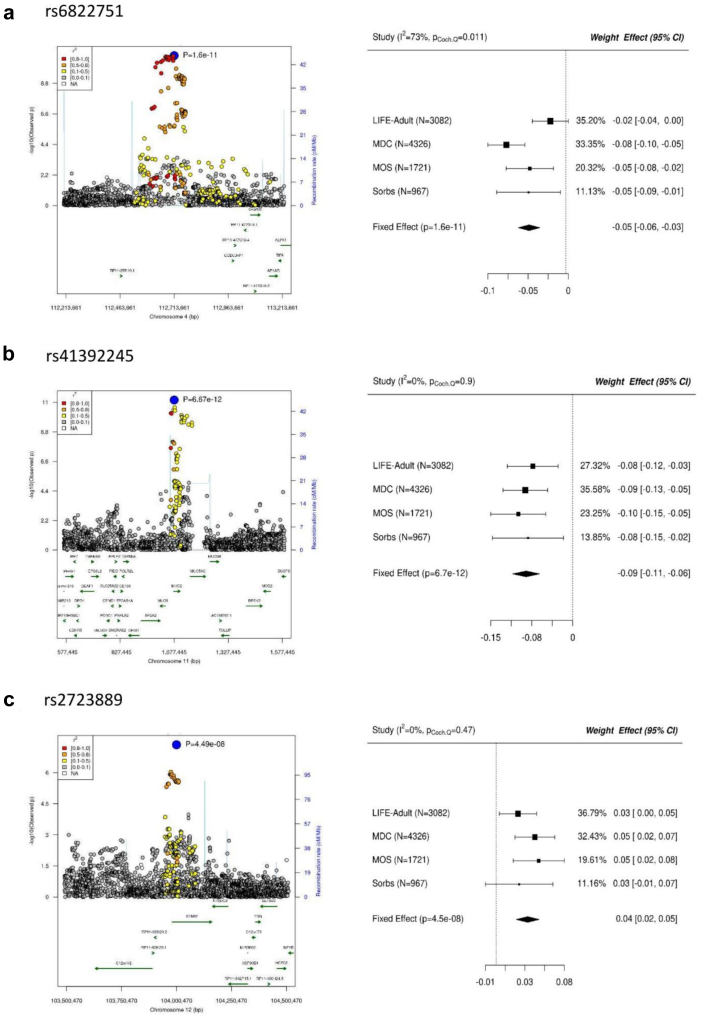
**Regional association (RA) and forest plots of three top-hit for pro-neurotensin.** On the left hand side, the RA and on the right hand side, the forest plots of the three independent top SNPs (a) rs6822751, b) rs41392245, and c) rs2723889) are shown. CI = confidence interval; Heterogeneity I-squared in meta-analysis; MDC = Malmö Diet Cancer cohort; MOS = Malmö Offspring Study; N = number; SNPs = single nucleotide polymorphisms.

The prospective GWAMA in 10,096 participants revealed 78 genome-wide significant hits distributed within three loci at 4q25, 11p15.5, and 12q23.3 (see Fig. 2 for Manhattan plot, Fig. 3 for regional association plots of independent variables, Table 2 for statistics of the variants, Supplementary Table S3 for an extended top-list, positions refer to GRCh37). No signs of general inflation of test-statistics were detected (Lambda = 1.02; Supplementary Figure S2). Besides the respective top-hits, COJO revealed no independent associations at all loci.

**Table 2:** Statistics and annotations of the identified independent SNPs of our GWAMA on pro-NT.

GWAMA (N = 10,096)								Meta-analysis of our GWAMA studies and Pietzner et al. (N = 20,804)		
SNP (effect/other allele)	cytoband/genomic position	Proposed candidate gene (distance)	P-value	beta	SE	I^2^	Explained variance	P-value	beta	SE
rs6822751 (T/G)	4q25/112,713,661		1.6 × 10^−11^	−0.047	0.007	0.73	0.004	9.3 × 10^−18^	−0.054	0.006
rs41392245 (T/C)	11p15.5/1,077,445	*MUC2* (0 kb)	6.7 × 10^−12^	−0.088	0.013	0	0.005	1.7 × 10^−16^	−0.094	0.012
rs2723889 (C/T)	12q23.3/104,000,470	*STAB2* (0 kb)	4.5 × 10^−08^	0.037	0.007	0	0.0030	2.3 × 10^−10^	0.039	0.006

Association Statistics (two-sided Z-test) are shown. We also provide statistics for combining our study with that of Pietzner et al.^31^ using semi-quantitative pro-NT measurements. Beta estimates correspond to log-changes of pro-NT levels. Genomic position is reported for GRCh37.

SNP = single nucleotide polymorphism; pro-NT = pro-neurotensin; GWAMA = genome-wide association meta-analysis; SE = standard error; I^2^ = Heterogeneity I-squared in meta-analysis.

4q25: The strongest association was observed for rs6822751 (G/T) (minor allele frequency (MAF) = 30%, beta = −0.047, P = 1.6 × 10^−11^, two-sided Z-test) (Fig. 3a). Across study heterogeneity is I^2^ = 73%. All studies showed the same trend but there was a stronger variance of effect sizes. In meta-regression analysis accounting for ethnicity (MR-MEGA), no signs of ancestry heterogeneity were detected (P_anc_ = 0.18, $\chi^{2}$ test) and the top variant remains genome-wide significant in this analysis (see Supplementary Table S4).

An extended meta-analysis of our index SNP also incorporating statistics derived from semi-quantitative proteome analysis of Pietzner et al. (N = 10,708)^31^ corroborated our findings (Table 2, Supplementary Table S5). The 99% credible set of the top-hit contained 41 variants (Supplementary Table S6). The locus appears to be pleiotropic with LD to other GWAS hits with BMI among them (Supplementary Table S3). The strongest associated variants are in a region without genes but there are several genes and eQTL cis-effects in the same haploblock. Based on these data, a possible involvement of a non-coding RNA as well as several *cis*-regulated genes (e.g. *ALPK1* and a *trans*-effect *CAB39L*) could be considered as candidate genes. However, no co-localisations between the pro-NT hit and the respective eQTL hits could be detected (Supplementary Table S7), and gene-expression association analysis based on MetaXcan did also not provide supportive evidence for any of these genes (Supplementary Table S8). Several mQTL colocalisations were observed at this locus comprising the genes *CCDC109B*, *MMAA*, *ZCCHC4*, *CTSO*, and *CDKL2* (Supplementary Table S9). Although relationships of these genes to pro-NT are conceivable, no clear candidate gene could be assigned to this locus.

11p15.5 (*Mucin 2* (*MUC2)*): The strongest association was observed for rs41392245 (C/T) (MAF = 7.2%, beta = −0.088, P = 6.7 × 10^−12^, two-sided Z-test) (Fig. 3b). No signs of across study heterogeneity were detected (I^2^ = 0%). Accordingly, all four studies showed consistent and significant effects and no signs of ancestry heterogeneity were observed (P_anc_ = 0.70, $\chi^{2}$ test; Supplementary Table S4). Meta-analysis including the data of Pietzner et al^31^ further strengthened the findings (P = 9.3 × 10^−18^, two-sided Z-test, Table 2, Supplementary Table S5). The 99% credible set comprises 22 variants, while the top-associated variant shows 67% PP of being causal (Supplementary Table S6). The variant is not in LD with published GWAS variants. Most of the variants of the credible set map within or near *MUC2* and the top-SNP itself is in the intron of *MUC2*. There is also an associated variant rs2856111 of the credible set with a strong CADD score^27^ of 22.6 in *MUC2* (Supplementary Table S3) representing an exon missense mutation. Co-localisations with mQTLs of *MUC2* were also observed (Supplementary Table S9). Accordingly, we consider *MUC2* as the most plausible candidate gene at this locus.

12q23.3 (*Stabilin 2* (*STAB2*)): Finally, at 12q23.3 we observed the strongest association for rs2723889 (T/C) (MAF = 32%, beta = 0.037, P = 4.5 × 10^−8^, two-sided Z-test) (Fig. 3c) with no signs of heterogeneity between studies (I^2^ = 0) and no ancestry heterogeneity (P_anc_ = 0.24, $\chi^{2}$ test; Supplementary Table S3). Combined meta-SNP analysis including the semi-quantitative association statistics supported the finding (P = 2.3 × 10^−10^, two-sided Z-test, Table 2, Supplementary Table S5). The 99% credible set of the top hit contains 210 variants (Supplementary Table S6). The top-variant is located in the coding region of *STAB2* and there is also a strong support for co-localisation of the pro-NT signal with a *STAB2* eQTL in Heart Atrial Appendage tissue (Supplementary Table S7). Moreover, co-localisations with mQTLs of *STAB2* were also observed (Supplementary Table S9). MetaXcan analysis provided support that the gene-expression of *STAB2* in colon traverse, colon sigmoid and whole blood is associated with pro-NT (all gene-wise q-values <1.6%; Supplementary Table S8). Thus, *STAB2* is the most plausible candidate here.

We further performed analyses of variants near the *NT* gene-body. No nominally significant SNPs were identified, in particular, we could not replicate the finding of Sentinelli et al.^6^ regarding association of rs2234762 with pro-NT. Gene-based association analysis by MetaXcan also revealed no associations of the *NT*-gene-expression (Supplementary Table S8). The regional association plot of the NT locus is shown as Supplementary Figure S3.

### Pro-NT loci are independent of BMI

Due to the known relationship between pro-NT and BMI, we examined this issue in more detail. Based on the LIFE-Adult study, we confirmed a significant association between pro-NT and BMI (β = 5.6 × 10^−3^, P = 1.5 × 10^−3^ in a linear regression analysis adjusting for age and sex). To exclude any secondary effects mediated by BMI, we calculated conditional association statistics using publicly available summary statistics. All associations remained genome-wide significant after conditioning on BMI (see Supplementary Table S10).

### Pro-NT is linked to lipid metabolism

GO enrichment analysis of the top 5% genes most highly correlated with *NTS* in human small intestine (GTEx V7 database) revealed a significant overrepresentation of genes involved in lipid metabolism (P = 1.8 × 10^−19^; one-tailed Fisher's exact test). This enrichment appeared to be driven largely by the strong co-expression between *NTS* and multiple apolipoprotein (APO) genes.

In *Nts*-knockout mice, we identified 314 DEGs under nutrient-rich conditions and 621 under fasting conditions, compared to controls. GO analysis of these DEGs consistently highlighted pathways related to lipid metabolism. Specifically, nutrient-rich conditions showed enrichment for lipid metabolic processes, while fasting conditions indicated activation of lipid biosynthesis and broader regulation of lipid metabolism.

Comparative analysis showed a significant overlap between the *NTS*-correlated human genes and the DEGs in mice: 29 genes overlapped with the nutrient-rich condition (P = 1.6 × 10^−6^, one-tailed Fisher's exact test) and 105 genes with the fasting condition (P = 2.6 × 10^−39^, one-tailed Fisher's exact test). Eleven genes were shared across all three datasets (human correlated genes, and both mouse conditions), including *CDA*, *MALRD1*, *ENTPD5*, *ARG2*, *CES2*, *SLC2A5*, *NDRG1*, *ALPI*, *PBLD*, *DPEP1*, and *SLC28A1*.

Notably, *APOB*—a key gene in lipid transport—was upregulated in *Nts*-knockout mice during fasting and showed a strong positive correlation with *NTS* expression in human intestine (r = 0.78).

Despite these consistent molecular associations, no direct phenotypic correlations were observed between plasma pro-NT levels and standard lipid parameters (HDL, LDL, TG, total cholesterol) in either the LIFE-Adult or Sorbs cohorts. Moreover, no significant genetic correlations were identified between pro-NT and these lipid traits (Table 3, Supplementary Table S11). Thus, although transcriptomic data suggest a role for pro-NT in lipid metabolism at the intestinal level, our cross-sectional analyses do not support a direct regulatory effect of pro-NT on plasma lipid concentrations, in contrast to previous claims in the literature.^2^

**Table 3:** Genetic correlation analysis of pro-neurotensin with brain volumes.

Correlated trait	Heritability estimate	Genetic correlation with pro-NT	SE	P-value
Amygdala	0.097	−0.137	0.242	0.573
Brainstem	0.327	−0.152	0.137	0.268
Caudate nucleus	0.305	−0.012	0.138	0.933
Globus pallidus	**0.171**	−**0.442**	**0.198**	**0.026**
Nucleus accumbens	0.190	−0.209	0.166	0.207
Putamen	0.297	−0.181	0.151	0.230
Thalamus	0.167	−0.214	0.183	0.242

Linkage disequilibrium (LD) score regression was conducted to estimate the correlation of pro-neurotensin and brain structure phenotypes^39^ using the tool LDSC v1.0.1.^37^^,^^38^ Significantly correlated traits with P < 0.05 are highlighted in bold (P-values calculated with a two-sided Z-test). The whole outcome of the analysis is provided in Supplementary Table S11.

SE = standard error of genetic correlation.

### Pro-NT is linked to various brain phenotypes

GCTA Analysis: Since NT is a neurotransmitter, we also investigated the link of pro-NT with gene expression patterns in the brain. GCTA addressing tissue-specific gene expression profiles available in GTEx V7 uncovered genes which correlated with *NTS* expression at different brain areas in humans (mediodorsal nucleus of thalamus; grey matter of forebrain; posteroventral (interior) parietal cortex; posterior (caudal) superior cortex; primary visual cortex (striate cortex, area V1/17; dorsolateral prefrontal cortex; neocortex (isocortex))). The top 5% of genes with highest correlations with *NTS* showed consistent patterns through different developmental stages (prenatal, infant, and adult)^46^ especially in the mediodorsal nucleus of the thalamus (Supplementary Table S12).

Genetic Correlation and Mendelian Randomisation Analysis: Based on LD-Score regression, we estimated the heritability of pro-NT by 10.9% (P = 0.031, one-sided Z-test). Among the considered subcortical brain phenotypes, we detected a significant genetic correlation of pro-NT with globus pallidus volume (r_g_ = −0.44, P = 0.026; two-sided Z-test) (Table 3, Supplementary Table S13). Thus, this trait was subjected to MR analysis aiming at clarifying a potential causal relationship of pro-NT with globus pallidus volume. We performed MR analysis using the three independent SNPs from our GWAMA as instruments of pro-NT concentrations. Indeed, we detected a causal effect of pro-NT on globus pallidus volume (beta_IVW_ = 0.253, P = 0.029; two-sided Z-test), which showed no sign of heterogeneity (Q = 1.25, P(Q) = 0.53, two-sided Z-test). In sensitivity analyses using methods robust against violations of MR conditions, the causal effect was significant in the penalised, robust and penalised-robust IVW (e.g. beta_IVW,p,r_ = 0.257, P = 0.001, two-sided Z-test), but not in the simple MR-Egger (beta_Egger_ = 0.480, P = 0.196, two-sided Z-test). However, in the robust and penalised-robust MR-Egger, the effect again reached significance (P = 7.4 × 10^−5^, two-sided Z-test). In all MR-Egger approaches, the estimate of the intercept was not significantly different from zero, indicating no relevant pleiotropic effect.

The opposite directions observed in our genetic correlation and MR analyses can be explained by the different genetic signals they capture. Genetic correlation reflects the average shared genetic architecture across the genome and is therefore influenced by widespread pleiotropic effects. In contrast, MR isolates the effects of a specific set of instrumental variants that are presumed to act along a causal pathway.

In our case, the genome-wide genetic correlation between pro-NT and globus pallidus volume is negative, whereas the MR instruments represent a small subset of variants showing a positive relationship. This discrepancy is illustrated in the Z-score plot (Supplementary Figure S4), where most variants follow a negative trend but the MR instruments appear as positive outliers. Such a pattern can arise when horizontal pleiotropy shapes the genome-wide correlation, while the causal signal captured by the MR instruments points in the opposite direction.

Further details of MR results and the single SNP statistics are presented in Supplementary Table S13 and Supplementary Table S2.

### MRI-based connectivity of the reward network analysis supports neural impact of pro-NT

To better understand the observed genetic correlation of pro-NT with various brain phenotypes, we investigated whether the SNPs associated with pro-NT were related to the connectivity of the reward network which had been previously found to be associated with obesity.^9^^,^^10^ Respective data were available for a subset of our LIFE-Adult cohort (N = 1090). Except for NOS CC (P_uncorr_ = 0.16), simple linear regressions adjusted for age and sex confirmed that a higher BMI correlates to less reward network coherence, i.e. to lower FA CS, NOS CS, and FA CC (all t < −4.2, all P < 3.4 × 10^−5^).

**Fig. 4:**
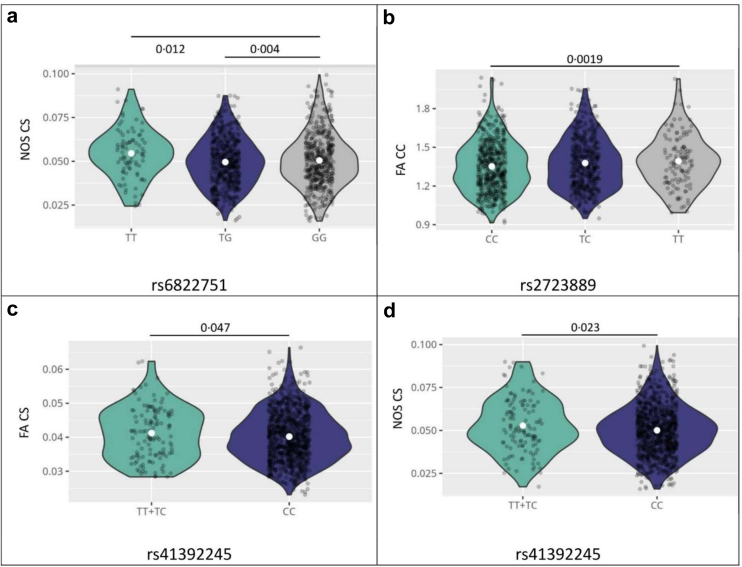
**Associations of top hit genetic variants with traits of the reward network in 1090 subjects of the LIFE-Adult cohort.** a) shows the association of rs6822751 and NOS CS; b) presents rs2723889 and FA CC. The association of rs41392245 with FA CS is shown in c) and with NOS CS in d). NOS = total number of connecting streamlines touching both regions; FA = mean fractional anisotropy; CS = connectivity strength; CC = clustering coefficient.

4q25 locus: For rs6822751 the additive genotype model of the effects of the risk alleles was not significant (all P > 0.19, linear regression) but for the genotype groups, NOS CS was significantly higher for participants carrying pro-NT decreasing T-alleles compared to non-carriers (one T-allele: beta = 0.0047, SE = 0.0016, P = 0.004, linear regression; two T-alleles: beta = 0.0041, SE = 0.0016, P = 0.012; linear regression adjusted for age and sex) (Fig. 4a). No other associations with FA CS or FA or NOS CC reached significance for this genotype.

11p15.5–*MUC2* locus: For the two rs41392245 C:T genotype groups, we observed that participants with pro-NT decreasing genotypes TT exhibited significantly higher FA CS (beta = 0.0013, SE = 0.0006, P = 0.047, linear regression corrected for age and sex) (Fig. 4c), and higher NOS CS (beta = 0.003, SE = 0.0013, P = 0.023, linear regression corrected for age and sex) (Fig. 4d), compared to carriers of one T-allele. This association was similar for NOS CS (P = 0.029, linear regression) and somewhat attenuated for FA CS (P = 0.08, linear regression) when additionally correcting for BMI in the models. No associations were found for FA CC and NOS CC for this genotype.

12q23.3–*STAB2* locus: The additive genotype model indicated that carrying pro-NT decreasing rs2723889 TT-alleles (12q23.3) related significantly to higher FA CC (beta = 0.021, SE = 0.008, P = 0.0019, linear regression corrected for age and sex) (Fig. 4b). The association did not change when adding BMI to the model (beta = 0.020, SE = 0.008, P = 0.017, linear regression).

All results of the analysis are shown in Supplementary Figure S5.

None of the whole-brain metrics were associated with the genotypes (all P > 0.05).

In sum, these results indicate lower reward network coherence in alleles associated with higher pro-NT.

## Discussion

NT is a tridecapeptide with a wide range of pleiotropic functions. It is involved in the control of lipid homoeostasis, blood pressure regulation, and body weight control and the development of mood disorders.^1^^,^^47^, ^48^, ^49^ Despite its broad spectrum of physiological effects, the underlying mechanisms of action are not fully understood.

In this study, we investigated the genetic factors influencing alterations in circulating peripheral concentrations of pro-NT. We conducted a prospective GWAMA involving 10,096 participants and identified three genetic loci. It must be acknowledged that potential bias such as selective non-participation of studies, incomplete data submission or selective reporting within datasets can affect the outcome of a prospective meta-analysis.

By bioinformatic analyses, we could assign clear candidate genes to two of these loci. At the locus 11p15.5 (rs41392245) we identified *MUC2*, which encodes a high molecular weight glycoprotein produced by various epithelial tissues. There are two different types of mucins: the classical gel-forming mucins (like among others MUC2) and the second transmembrane mucins.^50^ MUC2 is the major component of the intestine mucus layer forming extremely large polymers. It is present in the small and large intestine, forming the mucus skeleton.^51^ Furthermore, MUC2 enhances gut homoeostasis and oral tolerance by influencing dendritic cells and intestinal epithelial cells.^52^ Controlling digestion and absorption of ingested nutrients and the maintenance of the intestinal stem cell function under starvation could be one possible mechanism explaining NT's peripheral and central role in body weight management.^1^^,^^53^^,^^54^ Additionally, the role of MUC2 in maintaining a healthy microbiota, which is vital for intestinal homoeostasis, also aligns with NT's influence on body weight balance.^55^ A link between neuropeptides and mucins has recently been established by showing that MUC2 can transmit immunomodulatory signals to dendritic cells and thus facilitate the acquisition of immune tolerance in the intestinal mucosa.^52^^,^^56^

Another associated locus was 12q23.3 (rs2723889) with the candidate gene *STAB2*. *STAB2* encodes a transmembrane receptor involved in angiogenesis, lymphocyte homing, cell adhesion, and receptor scavenging. The protein is primarily expressed on sinusoidal endothelial cell of the liver, spleen, and lymph node.^57^ The STAB2 transmembrane receptor, belonging to a “superfamily” of scavenger receptors, is the only scavenger receptor binding hyaluronan, but also binds and internalises modified LDL-chol, and a variety of ligands, including endogenous proteins and pathogens.^58^^,^^59^ Moreover, STAB2 also acts as a primary systemic scavenger receptor for heparin, chondroitin sulphate, dermatan sulphate, non-glycosaminoglycan, acetylated and oxidised LDL-chol, pro-collagen pro-peptides and advanced glycation end products.^60^^,^^61^ Thus, STAB2 contributes to the clearance of circulating plasma molecules controlling distant organ homoeostasis. This might partially explain the strong *cis*-effects in several tissues like adipose tissue, oesophagus, lung, skin, and thyroid identified for *STAB2* and the signal within our MetaXcan analysis for effects of *STAB2* gene expression in whole blood and colon on NT. The association of pro-NT with *MUC2*, a major component of the intestinal mucus layer, may explain the effect of *STAB2* in the colon.

Important to mention here, the strongest genetic association was found at 4q25, but no clear candidate could be assigned here. Thus, this locus warrants further investigations.

We also support the relationship between pro-NT and gut as well as lipid metabolism, by gene expression analysis in intestinal mucosal layers and those involved in microbiota protection and post-translational processing. Moreover, pro-NT itself undergoes site-specific post-translational processing, resulting in distinct transcripts that may also act centrally.^62^^,^^63^ Due to the reported pleiotropic role of neurotensin involving a wide range of physiological functions in both, the central and peripheral system, we addressed its role in the gut-brain axis. The gut-brain axis plays a crucial role in lipid metabolism, with the gut microbiota influencing brain lipid composition^64^ and a wide range of brain regions and tissues holding different components of the neurotensin system (reviewed in Gereau et al.^65^). Based on GTEx data, we found genes correlating with *NTS* expression in brain to be linked to the size of the thalamus, particularly the mediodorsal nucleus, which is involved in executive functions like planning, cognitive control, working memory, and decision-making due to its connections with the prefrontal cortex.

We observed a negative genetic correlation between pro-NT and globus pallidus, which is a structure in the basal ganglia and primarily known for its role in movement control and inhibition of involuntary movement.^66^ We also observed a positive causal relationship between pro-NT and globus pallidus volume identified by MR analysis. In the LDSC analysis, we detected a significant genetic correlation of pro-NT with globus pallidus volume but MR analysis showed a significant and consistent causal association with globus pallidus volume in an opposite direction. The opposite directions highlight an important conceptual distinction. Genetic correlation reflects the average genome-wide sharing of genetic effects and can be influenced by horizontal pleiotropy, whereas MR focuses on the effects of specific instrumental variants presumed to act causally. Consequently, if the instrumental SNPs represent a subset of variants whose effects differ from the broader genetic background, discordant directions between genetic correlation and MR may arise. Animal studies suggest that the globus pallidus also plays a role in reward processing, e.g. due to its connection to the lateral habenula which in turn affects dopaminergic and serotonergic systems.^67^ Even though the globus pallidus is not part of the key dopaminergic reward network, formed by the ventral striatum, substantia nigra and its projections to orbitofrontal cortices,^8^ it is plausible it plays a role in reward processing.^68^

Regarding reward network analysis, pro-NT decreasing T-allele carrier of all three genetic top hits presented significant higher reward network connectivity, based on number and strength of connections. This phenotype is of interest due to the known negative correlation of reward network connectivity with BMI,^9^ which may contribute to obesity and related metabolic disorders.^69^ While NT has been considered to not pass the blood-brain-barrier (BBB) and peripheral vs. central effects of NT injections induce differential effects,^70^^,^^71^ peripheral pro-NT may still directly or indirectly affect central nervous function via body-brain interactions through nerval, hormonal, immune-related, or metabolic routes such as short chain fatty acid alterations.^72^ NT, a peptide predominantly found in entero-endocrine cells of the small bowel, plays a crucial role in facilitating fatty acid translocation and has implications in obesity.^73^ In addition, it could be speculated that peripheral pro-NT levels also reflect central pro-NT levels, indicating the possibility of parallel effects of genetic predisposition on brain pro-NT and related NT level variability. NT has shown to serve a modulatory role of diverse neuronal functions in the brain, including reward-related behaviour implicated in feeding and motivated behaviour (e.g. ^74^^,^^75^, for review see^48^^,^^76^). Optogenetic studies, for example, suggested that NT-expressing neurons in subcortical areas signal social reward-related cues in female mice.^77^

The study presents a well-powered, prospective meta-analysis integrating genetic, transcriptomic, and neuroimaging evidence to clarify determinants of circulating pro-NT and its potential effects on brain structure. Its strengths include the use of multiple large cohorts, rigorous GWAS methodology, careful locus annotation, and complementary analyses such as Mendelian Randomisation and MRI-based network evaluation, all of which together support biologically coherent conclusions. However, some limitations remain—notably the inability to assign a clear candidate gene to the strongest locus, the moderate heterogeneity at 4q25, and the reliance on cross-sectional imaging data that restrict causal interpretation of neural findings. Additionally, parts of the mechanistic interpretation (e.g. links between peripheral pro-NT and central reward circuitry) remain speculative and would benefit from more direct functional validation. Larger sample sizes are required to identify more genetic variants of the trait, to investigate interaction effects and to improve power for Mendelian Randomisation Analysis.

### Conclusion

In summary, our study provides new candidate genes of NT regulation and evidence for a causal relationship between pro-NT and brain structures, particularly the pallidum volume, suggesting a central action of pro-NT in movement activation and complex feedback mechanisms but further studies are warranted to further elucidate these mechanisms.

## Contributors

A. Tönjes, P. Kovacs and M. Scholz attributed to the conceptualisation; J. Breitfeld, K. Horn, P. Kovacs, M. Scholz, and A. Tönjes analysed data and wrote manuscript; A. Velluva, D. Le Duc, F. Beyer, J. Pott, and V. Witte analysed and validated the data; R. Baber is responsible for the LIFE biobank, M. Stumvoll, and A. Tönjes are responsible for the Sorbs cohort; O. Melander and A. Giontella are responsible for MDC and MOS cohorts.

All authors read and approved the final version of the manuscript.

## Data sharing statement

The datasets used and/or analysed during the current study will be available on GWAS Catalogue after publication.

## Declaration of interests

The authors declare that they have no competing interests.
