# Supplementary material for: Genetic dissection of serum pro-neurotensin suggests potential causal impact on brain structure
Source: eBioMedicine. 2026 Jan 10;124:106105. doi: 10.1016/j.ebiom.2025.106105 (PMC12818281; doi:10.1016/j.ebiom.2025.106105)
Supplement: Supplementary Figures [file mmc1.docx]

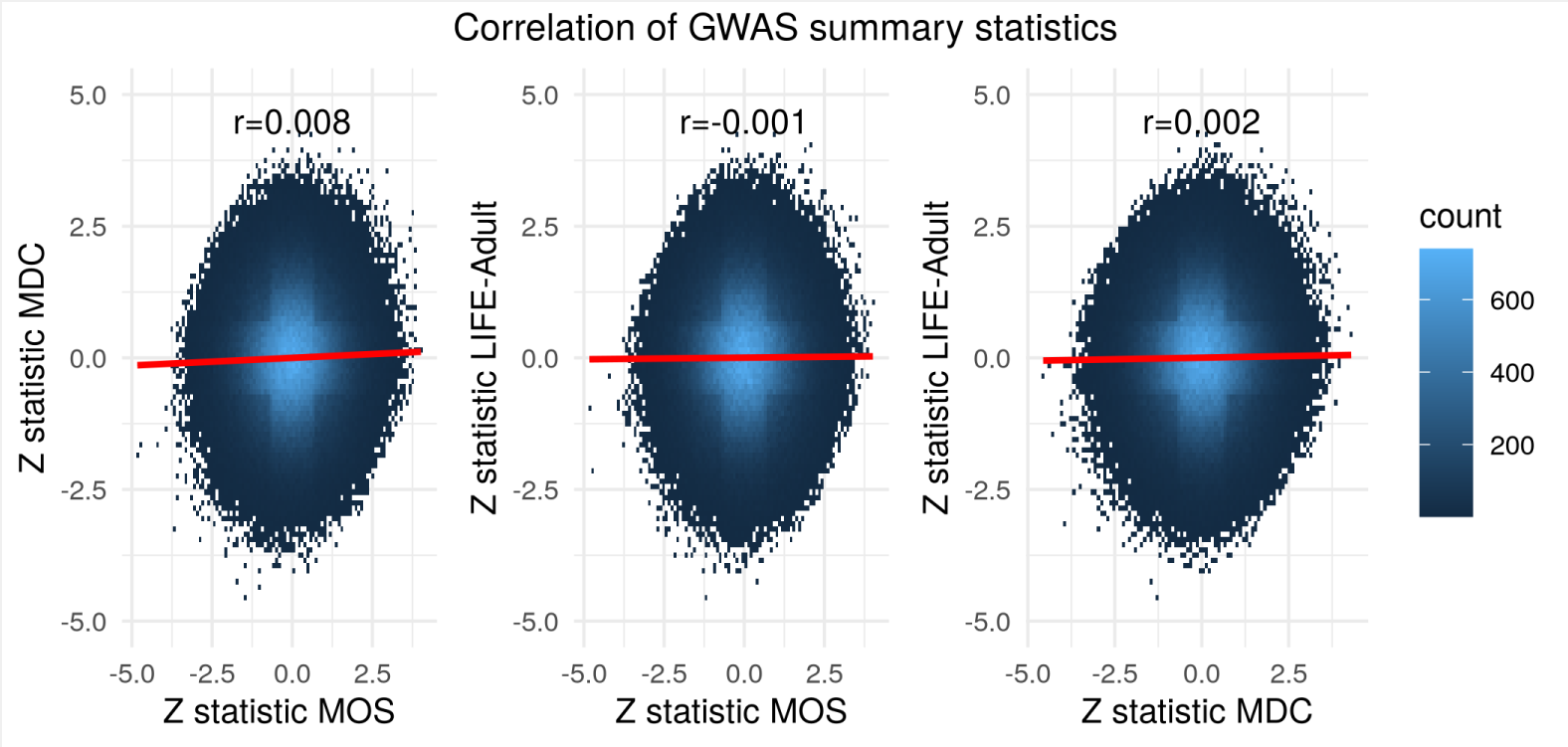


**Supplementary FigureS1**: **Correlation of GWAS summary statistics presents Z-scores between MDC, MOS and LIFE-Adult cohorts.** Blue squares represent 2D bins of SNP Z statistics generated using the function geom_bin_2d. The red line depicts the linear correlation estimate from geom_smooth(method=”lm”). The aforementioned functions belong to the R library ggplot2. The reported Pearson correlation coefficient (r) was calculated using the R function cor.test(method=”pearson”) from the stats library.

GWAS = genome-wide association study; MDC = Malmö Diet Cancer; MOS = Malmö Offspring Study


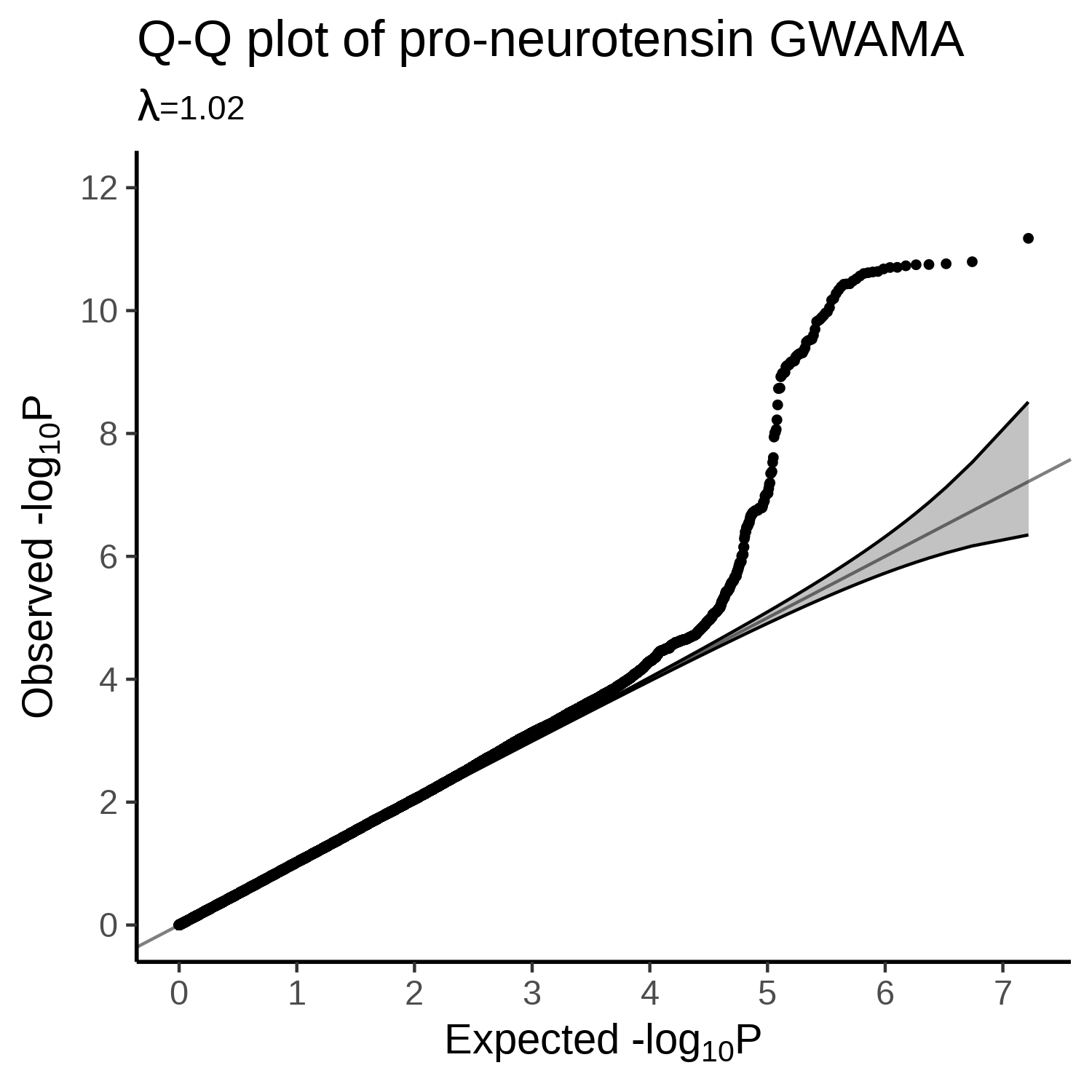


**Supplementary FigureS2: Q-Q plot of the pro-neurotensin meta-genome-wide association study (λ = 1.02).**


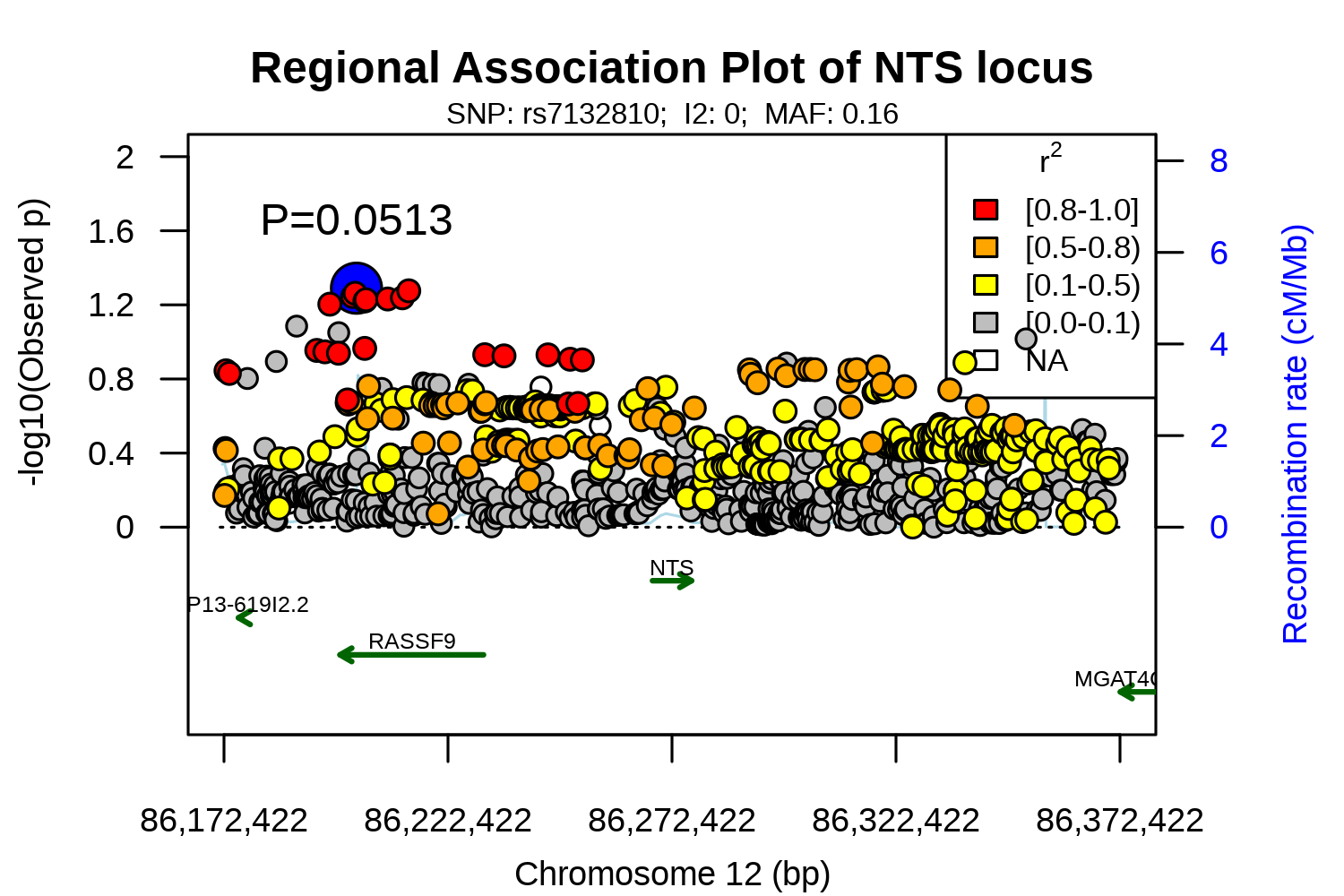


**Supplementary FigureS3: Regional association plot of the *neurotensin* locus on chromosome 12.**

Blue dot represents variant rs7132810 with I^2^ = 0 and minor allele frequency = 0.16. I^2^=Heterogeneity I-squared in meta-analysis

*
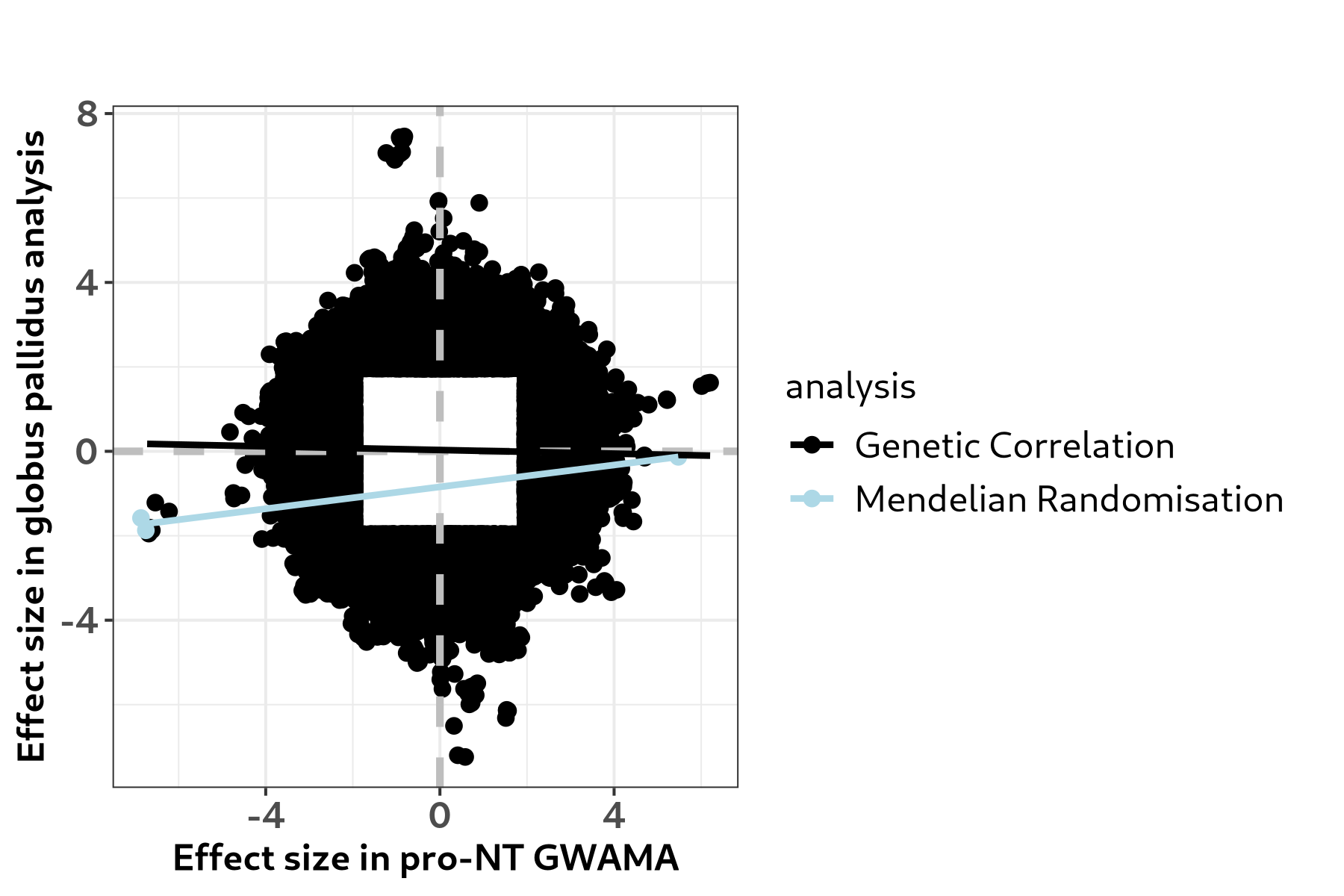
*

**Supplementary FigureS4: Z-score plot illustrating the opposite directions observed for genetic correlation and Mendelian Randomisation between pro-neurotensin and globus pallidus volume.** Black dots represent SNPs that were nominally significant in either analysis. These show a slight negative trend, which serves to visualise the general effect direction but does not represent the numerical value of the genetic correlation estimate. SNPs used as genetic instruments in our MR analysis are highlighted in blue and display a positive trend, illustrating the discordance between the genome-wide background signal and the causal effect direction. The black and blue line were generated with the function geom_smooth(method=lm’, se=FALSE) of the R library ggplot2 to further illustrate the different effect directions.

Pro-NT=pro-neurotensin; GWAMA=genome-wide analysis meta-analysis; SNPs=singel nucleotide polymorphisms ; MR=Mendelian Randomisation


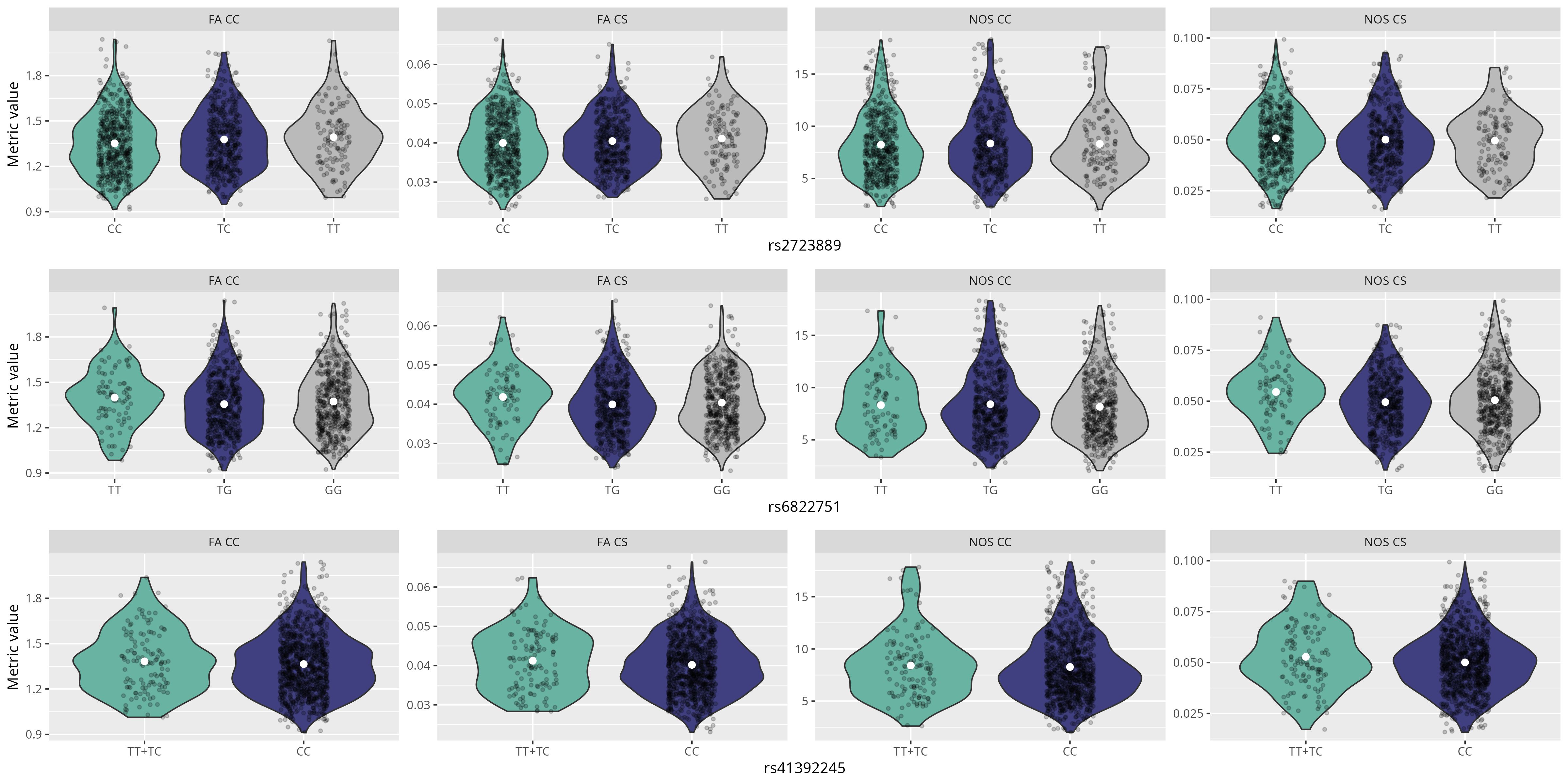


**

*

**

*

*

**Supplementary FigureS5: Associations of all top hit genetic variants with all traits of the reward network in 1,090 subjects of the LIFE-Adult cohort.**

NOS = total number of connecting streamlines touching both brain regions; FA = mean fractional anisotropy; CS = connectivity strength, CC = clustering coefficient; */**=P-value<0·05/0·01
