# Supplementary material for: Genetic dissection of serum pro-neurotensin suggests potential causal impact on brain structure
Source: eBioMedicine. 2026 Jan 10;124:106105. doi: 10.1016/j.ebiom.2025.106105 (PMC12818281; doi:10.1016/j.ebiom.2025.106105)
Supplement: Supplementary Table 12 [file mmc13.docx]

**Supplementary TableS12:** Allen Brain Atlas (ABA) Enrichment of top 5% highest correlating genes in the brain.

| **Age category** | **Structure ID in ABA** | **Structure** | **N of annotated genes** | **Mean FWER** |
| --- | --- | --- | --- | --- |
| Prenatal | 10398 | MD-mediodorsal nucleus of thalamus | 489 | <0.0001 |
| Infant | 10157 | FGM-grey matter of forebrain | 572 | 0.0001 |
|  | 10398 | MD-mediodorsal nucleus of thalamus | 489 | <0.0001 |
| Child | 10157 | FGM-grey matter of forebrain | 572 | 0.0004 |
|  | 10398 | MD-mediodorsal nucleus of thalamus | 489 | 0.0104 |
| Adolescent | 10225 | IPC-posteroventral (interior) parietal cortex | 362 | 0.0241 |
|  | 10243 | STC-posterior (caudal) superior cortex | 335 | 0.036 |
| Adult | 10157 | FGM-grey matter of forebrain | 572 | 0.0003 |
|  | 10269 | V1C-primary visual cortex (striate cortex, area V1/17) | 365 | 0.0003 |
|  | 10398 | MD-mediodorsal nucleus of thalamus | 489 | <0.0001 |
|  | 10173 | DFC-dorsolateral prefrontal cortex | 358 | 0.0273 |
|  | 10160 | NCx-neocortex (isocortex) | 408 | 0.038 |

ABA=Allen Brain Atlas (<https://portal.brain-map.org>); ID=identity; N=number; FWER= family-wise error rate
